# ReRF-Pred: predicting amyloidogenic regions of proteins based on their pseudo amino acid composition and tripeptide composition

**DOI:** 10.1186/s12859-021-04446-4

**Published:** 2021-11-09

**Authors:** Zhixia Teng, Zitong Zhang, Zhen Tian, Yanjuan Li, Guohua Wang

**Affiliations:** 1 College of Information and Computer Engineering, Northeast Forestry University, Harbin, 150040 China; 2 College of Information Engineering, Zhengzhou University, Zhengzhou, 450001 China; 3 College of Electrical and Information Engineering, Quzhou University, Quzhou, 324000 China

**Keywords:** Amyloid, Tripeptide composition, PseAAC, Binomial distribution, Random forest

## Abstract

**Background:**

Amyloids are insoluble fibrillar aggregates that are highly associated with complex human diseases, such as Alzheimer’s disease, Parkinson’s disease, and type II diabetes. Recently, many studies reported that some specific regions of amino acid sequences may be responsible for the amyloidosis of proteins. It has become very important for elucidating the mechanism of amyloids that identifying the amyloidogenic regions. Accordingly, several computational methods have been put forward to discover amyloidogenic regions. The majority of these methods predicted amyloidogenic regions based on the physicochemical properties of amino acids. In fact, position, order, and correlation of amino acids may also influence the amyloidosis of proteins, which should be also considered in detecting amyloidogenic regions.

**Results:**

To address this problem, we proposed a novel machine-learning approach for predicting amyloidogenic regions, called ReRF-Pred. Firstly, the pseudo amino acid composition (PseAAC) was exploited to characterize physicochemical properties and correlation of amino acids. Secondly, tripeptides composition (TPC) was employed to represent the order and position of amino acids. To improve the distinguishability of TPC, all possible tripeptides were analyzed by the binomial distribution method, and only those which have significantly different distribution between positive and negative samples remained. Finally, all samples were characterized by PseAAC and TPC of their amino acid sequence, and a random forest-based amyloidogenic regions predictor was trained on these samples. It was proved by validation experiments that the feature set consisted of PseAAC and TPC is the most distinguishable one for detecting amyloidosis. Meanwhile, random forest is superior to other concerned classifiers on almost all metrics. To validate the effectiveness of our model, ReRF-Pred is compared with a series of gold-standard methods on two datasets: Pep-251 and Reg33. The results suggested our method has the best overall performance and makes significant improvements in discovering amyloidogenic regions.

**Conclusions:**

The advantages of our method are mainly attributed to that PseAAC and TPC can describe the differences between amyloids and other proteins successfully. The ReRF-Pred server can be accessed at http://106.12.83.135:8080/ReRF-Pred/.

**Supplementary Information:**

The online version contains supplementary material available at 10.1186/s12859-021-04446-4.

## Background

Amyloids are fibrillar aggregates generated from soluble proteins or peptides under certain conditions (eg. ionic strength, temperature, etc.). As known, the core of amyloid fibrils exhibits a cross-$\beta$ structure with the $\beta$-chains running perpendicular to the elongation axis of the fibrils [1]. Accordingly, multiple amyloid fibrils can aggregate to form amyloid protein which has the highly ordered steric zipper structure [2]. In recent years it is reported by many studies that amyloid proteins are closely associated with several human complex diseases including Alzheimer’s disease [3, 4], Parkinson’s disease [5], Huntington’s disease [6], familial Mediterranean fever [7], type II diabetes [8, 9], etc. It is inferred that amyloid proteins may provide new therapeutic targets for these diseases. Consequently, many efforts have been made in the field of identifying amyloid proteins.

At first, amyloid proteins only could be discovered by in vitro techniques such as observing their fiber structure with the electron microscope and X-ray, or Congo red and Thioflavin T staining method [10]. However, these in vitro methods are time-consuming and costly. Therefore, several computational methods have been developed to predict amyloid proteins. The computational methods can be roughly classified into sequence-based approaches and structure-based approaches. The first group includes Zyggregator [11], AGGRESCAN [12], Waltz [13], and FISH Amyloid [14], which utilized the site-specific or physicochemical properties of amino acids (e.g., hydrophobicity, solvent accessibility) to make predictions. The second group covers NetCSSP [15], PASTA [16], and FoldAmyloid [17], which focus on analyzing the cross-$\beta$ structure of amyloid fibrils or the 3D coordinates of protein atoms. Besides, some methods including AmylPred [18], AmylPred2 [19], and MetAmyl [20], improved the performance of prediction by assembling several different predictors.

Subsequently, machine learning-based methods were put forward to detect amyloid proteins. Família et al. [21] selected features recursively from seven physicochemical and biochemical properties of amino acids and employed feed-forward neural networks to estimate the amyloidosis probabilities of peptides and proteins. Burdukiewicz et al. [22] combined multiple physicochemical properties using n-grams to identify the amyloid proteins. Bouziane et al. [23] collected features on structural conformation and solvent accessibility, and constructed a model to predict amyloidogenic regions using the string kernel-based support vector machine (SVM). Zhou et al. [24] utilized position-specific scoring matrix (PSSM) and physicochemical properties including hydrophilicity, aggregation tendency, and packing density to develop an SVM-based predictor for amyloidogenic proteins. These methods make great progress in the field of predicting amyloid proteins. However, identifying amyloids is only a small step toward designing therapeutic targets, we still have not enough knowledge about the detailed mechanism of amyloidosis to develop therapeutic targets.

A lot of theoretical and experimental evidence illustrates that amyloidosis may be promoted and guided by one or more short and specific fragments of protein sequences, called hot spots [25, 26]. Therefore, to elucidate the detailed mechanism of amyloidosis, it is the fundamental step to identify the region which induces amyloidosis of protein. Although the methods proposed by Família et al. and Bouziane et al. can predict amyloidogenic regions, their performance still can be improved for the following reasons. Firstly, both of them ignored that the order of amino acids may also affect the amyloidosis of protein, just like physicochemical properties. For example, both “VVLL” and “VLVL” are hydrophobic peptides, but they may differ in structure and function because of their completely different arrangements of amino acids. Secondly, plenty of studies suggested that the interaction between amino acids influences the mechanism of protein, which also did not be considered by these methods.

**Fig. 1:**
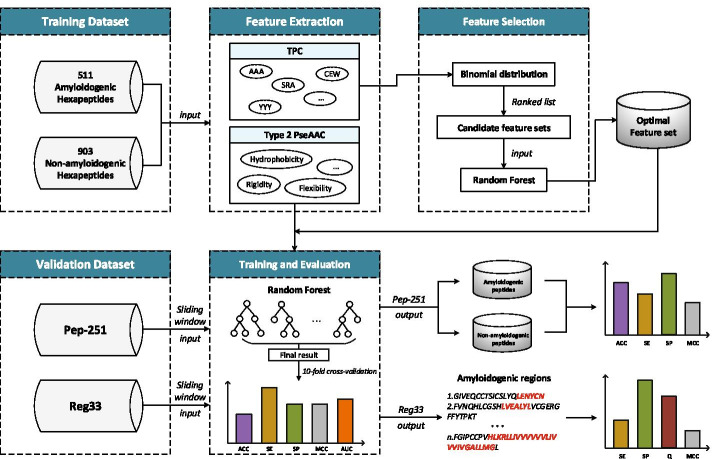
The frame chart of the ReRF-Pred

To address above mentioned issues, as displayed in Fig. 1, we proposed a novel prediction method for discovering amyloidogenic regions, named ReRF-Pred. First of all, the pseudo amino acid composition (PseAAC) was extracted to characterize physicochemical properties and correlation of amino acids. Next, tripeptides composition (TPC) was exploited to describe the order and position of amino acids. As known, a protein may be composed of 20 amino acids, which may form 8000 tripeptides. If all tripeptides are used as features, it will make the model computationally intensive and poorly interpreted. And then, to avoid this situation, the tripeptides with high contribution to locating amyloidogenic regions were selected through the binomial distribution method and utilized together with PseAAC to train the prediction model. Eventually, a random forest-based prediction model was trained on the hexapeptides of protein sequences because hexapeptides are the commonest form of amyloidogenic regions [27]. The details of our novel method will be illustrated in the following sections.

## Materials and methods

### Datasets

For the development and evaluation of ReRF-Pred, the following three datasets were used (Additional file 1).

The first dataset consists of all hexapeptides from two reliable databases, WaltzDB 2.0 [28] and AmyLoad [29], with 511 experimentally determined amyloidogenic hexapeptides and 903 non-amyloidogenic hexapeptides. This dataset was used as the training set in our method.

The second dataset, named Pep-251, is a more general dataset consisting of peptides with different lengths. It was extracted from the dataset Pep424 [30], used here to evaluate hotspot-guided small peptides amyloidosis. Our approach assumes that the minimum length of a hot spot is six residues, and thus peptides with unclear category delineation and shorter than seven residues were removed from Pep424. Note that all hexapeptides in Pep424 are included in the training set. The final Pep-251 contains 79 positives for amyloidogenic and 172 negatives.

The third dataset called Reg33 [19] consists of 33 proteins from the amylome. Each protein is annotated with amyloidogenic regions from the literature, with 1260 hotspot residues and 6571 regular residues. This dataset was used to evaluate the performance of ReRF-Pred in predicting amyloidogenic regions.

### Feature extraction

Feature extraction is the most important step in building a machine learning model [31–33], and effective features will greatly improve prediction performance [34–37]. In the present study, the composition, physicochemical properties of amino acids and their order information in the sequence are significant and indispensable for characterizing amyloidogenic regions. At this point, many single feature extraction strategies with excellent performance become not applicable. Therefore, we proposed a new method for fusing multiple sequence information, which combines Type 2 PseAAC feature and TPC feature to represent hotspots.

#### Type 2 PseAAC

The pseudo amino acid composition (PseAAC) [38] is a classical feature extraction algorithm proposed by analyzing the physicochemical properties of amino acids and the global order information of sequences [39–42]. Type 2 PseAAC [43] is also called the series correlation type. In this method, amino acid properties are used to reflect sequence order effects due to their important role in protein folding, interactions with molecules, and catalytic mechanisms [44–47]. The Type 2 PseAAC web server already provides six amino acid properties, including hydrophobicity, hydrophilicity, Mass, pK1 (alpha-COOH), pK2 (NH3), and pI (at 25$^{\circ }$C). On this basis, we added three different properties: rigidity, flexibility, and irreplaceability. Thus, a protein sequence *P* is represented as:

1 $$\begin{aligned} \begin{aligned} P = {({P_1},{P_2},\ldots ,{P_{20}},{P_{20 + 1}},...,{P_{20 + \lambda }},\ldots ,{P_{20 + 8\lambda + 1}},\ldots ,{P_{20 + 9\lambda }})^T}, \end{aligned} \end{aligned}$$

where

2 $$\begin{aligned} {{P}_{u}}=\left\{ \begin{aligned}&\frac{{{f}_{u}}}{\sum \nolimits _{i=1}^{20}{{{f}_{i}}}+w\sum \nolimits _{j=1}^{9\lambda }{{{\theta }_{j}}}}\text { }(1\le u\le 20), \\&\frac{w{{\theta }_{u}}}{\sum \nolimits _{i=1}^{20}{{{f}_{i}}}+w\sum \nolimits _{j=1}^{9\lambda }{{{\theta }_{j}}}}\,(20+1\le u\le 20+9\lambda ), \end{aligned} \right. \end{aligned}$$

where $f_u$ is the occurrence frequency of the 20 amino acids in the protein; *w* is the weight factor, which is set to 0.7 in this paper; $\theta _j$ reflects the correlation factor between two residues, which can be calculated by the following equation:

3 $$\begin{aligned} \left\{ \begin{aligned}&{{\theta }_{1}}=\frac{1}{L-1}\sum \nolimits _{i=1}^{L-1}{H_{i,i+1}^{1}} \\&{{\theta }_{2}}=\frac{1}{L-1}\sum \nolimits _{i=1}^{L-1}{H_{i,i+1}^{2}} \\&\text { }..................\text { } \\&{{\theta }_{9}}=\frac{1}{L-1}\sum \nolimits _{i=1}^{L-1}{H_{i,i+1}^{9}} \\&\text { }..................\text { } \\&{{\theta }_{9\lambda \text {-}8}}=\frac{1}{L-\lambda }\sum \nolimits _{i=1}^{L-\lambda }{H_{i,i+\lambda }^{1}} \\&{{\theta }_{9\lambda \text {-7}}}=\frac{1}{L-\lambda }\sum \nolimits _{i=1}^{L-\lambda }{H_{i,i+\lambda }^{2}} \\&\text { }..................\text { } \\&{{\theta }_{9\lambda }}=\frac{1}{L-\lambda }\sum \nolimits _{i=1}^{L-\lambda }{H_{i,i+\lambda }^{9}} \\ \end{aligned} \right. (\lambda <L), \end{aligned}$$

where *L* represents the length of a sequence; $\lambda$ is the counted rank of the correlation along a protein sequence, and the value should be less than *L*; if $\lambda$=1, $\theta _j$ reflects the correlation between adjacent amino acids; if $\lambda$=2, $\theta _j$ reflects the correlation between amino acids with an interval of 1; $H_{i,j}^k$ is the physicochemical properties correlation function given by:

4 $$\begin{aligned} H_{i,j}^{k}={{h}^{k}}({{R}_{i}})\cdot {{h}^{k}}({{R}_{j}}), \end{aligned}$$

where $R_i$ and $R_j$ are the *ith* and *jth* amino acid residue in the sequence, respectively; $h^1(R_i)$, $h^2(R_i)$, $\ldots$ , $h^9(R_i)$ represents the values of nine properties of $R_i$, respectively. Note that before substituting the values of $h^k(R_i)$, they were all subjected to a standard conversion as following:

5 $$\begin{aligned} {{h}^{k}}({{R}_{i}})=\frac{h_{0}^{k}({{R}_{i}})-\sum \nolimits _{j=1}^{20}{h_{0}^{k}({{R}_{j}})/20}}{\sqrt{\sum \nolimits _{u=1}^{20}{{{\left[ h_{0}^{k}({{R}_{u}})-\sum \nolimits _{j=1}^{20}{h_{0}^{k}({{R}_{j}})/20} \right] }^{2}}/20}}},(1\le k\le 9), \end{aligned}$$

where $h^k_0$ is the original value of the *kth* amino acid property.

As we can see from the above equations, Type 2 PseAAC incorporates a large amount of sequence order information in the correlation factor through the physicochemical properties of amino acids, which is extremely beneficial for representing amyloidogenic fragments.

#### Tripeptide composition

The tripeptide composition (TPC) method describes the position and order information of amino acids in a sequence [48, 49]. Li et al. [50] have confirmed that the TPC feature is beneficial for classifying amyloid proteins, and therefore we considered their utilization in the investigation of amyloidogenic regions. In this method, the occurrence frequencies of three consecutive amino acids in the sequence are used as the feature elements. The protein contains 20 native amino acids, and thus each sequence can be represented as a $20\times 20\times 20 = 8000$-dimensional feature vector:

6 $$\begin{aligned} F={{[{{f}_{1}},{{f}_{2}},\ldots ,{{f}_{8000}}]}^{T}}, \end{aligned}$$

where the frequency $f_i$ of the *ith* tripeptide can be calculated as:

7 $$\begin{aligned} {{f}_{i}}=\frac{{{N}_{i}}}{L-2}. \end{aligned}$$

where $N_i$ is the number of the *i*th tripeptide and *L* represents the length of a sequence.

### Feature selection

As mentioned above, we adopted the feature representation method of multi-information fusion, which mines the sequence information richly but brings more noise and redundant features [51–55]. In particular, the feature vector reaches 8000 dimensions in the TPC method, further screening of tripeptides that better represent hotspots is necessary. For the PseAAC feature, nine selected physicochemical properties were all retained considering their essential to reflecting the characteristics of amyloidogenic fragments. Therefore, we only discuss the selection and analysis of TPC features. In this study, the binomial distribution (BD) method [56, 57] was used for feature ranking.

By calculating the probability of the *ith* tripeptide in the class *j* samples, we can judge whether the occurrence of tripeptides in a certain kind of protein is random, like this:

8 $$\begin{aligned} {{P}_{ij}}=\sum \limits _{k={{n}_{ij}}}^{{{N}_{i}}}{\frac{{{N}_{i}}!}{k!\left( {{N}_{i}}-k \right) !}{{q}_{j}}^{k}{{\left( 1-{{q}_{j}} \right) }^{{{N}_{i}}-k}}}, \end{aligned}$$

where $q_j$ is the ratio of the number of tripeptides in class *j* samples to those in all samples, $n_{ij}$ and $N_i$ are the occurrence number of the *ith* tripeptide in class $j (j = 0,1)$ and all samples, respectively. If $P_{ij}$ is a small value, it indicates that the occurrence of tripeptides is deterministic. Hence, the confidence level (*CL*) of the *ith* tripeptide in the class *j* samples can be defined as:

9 $$\begin{aligned} C{{L}_{ij}}=1-{{P}_{ij}}. \end{aligned}$$

Obviously, each tripeptide feature has two *CL* values, and the larger one will be reserved. After calculating the confidence levels, we arranged the features in descending order by *CL* values to create a ranked list.

### Random forest

Ensemble learning is a hot topic in machine learning-related fields in recent years [58–60]. Its idea is to obtain better performance by combining the classification results of multiple single classifiers. The most effective ensemble learning algorithms are Bagging and Boosting, while Random Forest is a special Bagging algorithm whose base classifiers are *N* decision trees. Like Bagging, Random Forest is based on a bootstrap sampling technique, each time generate a new training set by randomly selecting *k* samples from the original training set with replacement. The difference is that random forest introduces attribute randomness, where the attributes of each node of the decision tree are generated from a small number of randomly selected attributes. Random forest was utilized in several bioinformatics researches [61–63].

In this study, we employed random forest as a classifier because it provides several unique advantages based on our data. The feature vectors extracted by the combined method of PseAAC and TPC belong to high-dimensional data, and the accuracy is not affected when random forest processing this type of data.

## Results and discussion

### Measurement

To evaluate and compare the performance of the model, we employed five metrics widely used in bioinformatics: accuracy (ACC), sensitivity (SE), specificity (SP), Q, and Mathew’s correlation coefficient (MCC) [64–67]. They are defined as follows:

10 $$\begin{aligned} ACC= & {} \frac{TP+TN}{TP+TN+FP+FN} \end{aligned}$$

11 $$\begin{aligned} SE= & {} \frac{TP}{TP+FN} \end{aligned}$$

12 $$\begin{aligned} SP= & {} \frac{TN}{TN+FP} \end{aligned}$$

13 $$\begin{aligned} Q= & {} \frac{SE+SP}{2} \end{aligned}$$

14 $$\begin{aligned} MCC= & {} \frac{TP\times TN-FP\times FN}{\sqrt{\left( TP+FP \right) \left( TP+FN \right) \left( TN+FP \right) \left( TN+FN \right) }}, \end{aligned}$$

where TP, FP, TN, and FN represent the number of true positive, false positive, true negative, and false negative, respectively. In the task of detecting amyloidogenic regions, TP, FP, TN, and FN are counted on a per residue basis. TP is the correctly predicted number of hotspot residues; FP is the number of regular residues predicted to be hotspot residues; TN is the correctly predicted number of regular residues; FN is the number of hotspot residues predicted to be regular residues. For example, given a segment of calcitonin CGNLSTCMLGTYTQDFNKFHTFPQTAIGVGAP, its experimentally verified hotspot region is DFNKFH (residues 15–20). If the predicted hotspot region is TYTQDFNKFHTFP (residues 11–23), then TP = 6, FP = 7, TN = 19, FN = 0. The SE and SP metrics measure the predictive ability of the model for positive and negative samples, respectively. The other three metrics, ACC, Q, and MCC, reflect the overall performance and stability of the model [68, 69]. Furthermore, receiver operating characteristic (ROC) curves are used to assess the real performance of the model more intuitively. We can quantitatively compare the decision-making ability of the models by calculating the area under the ROC curves (AUC) [70, 71]. For all the metrics mentioned above, the larger their values, the better performance the model has.

### Validating effectiveness of ReRF-pred

#### Comparison of different features

As previously stated, the amyloidogenicity of a protein may be represented by multiple sequence features. These features can be roughly divided into two groups: physicochemical properties-based features and sequence information-based features. The first group includes PseAAC, CTDC, CTDD, CTDT, and Conjoint Triad (CTriad). The second group covers Amino Acid Composition (AAC), Dipeptide Deviation from Expected Mean (DDE), Dipeptide composition (DPC), TPC, and BINARY. To verify the effectiveness of the proposed feature, we compared it with several other popular features. The 10-fold cross-validation results of ten single features and several combinations of features with good performance are listed in Table 1.

**Table 1:** Comparison of different features

	Features	ACC	SE	SP	MCC	AUC
*Mixed*	PseAAC+TPC	0.828	0.663	0.921	0.619	0.890
	PseAAC+AAC	0.825	0.671	0.911	0.611	0.887
	CTDD+DPC	0.818	0.716	0.875	0.600	0.882
*Physicochemical properties-based*	PseAAC	0.819	0.654	0.913	0.598	0.878
	CTDC	0.807	0.722	0.855	0.580	0.863
	CTDD	0.814	0.714	0.870	0.593	0.878
	CTDT	0.794	0.675	0.862	0.547	0.866
	CTriad	0.757	0.636	0.825	0.467	0.811
*Sequence information-based*	AAC	0.805	0.691	0.869	0.571	0.885
	DDE	0.803	0.687	0.868	0.566	0.884
	DPC	0.803	0.701	0.860	0.568	0.880
	TPC	0.801	0.628	0.898	0.556	0.873
	BINARY	0.787	0.652	0.864	0.530	0.866

As shown in Table 1, physicochemical properties-based features and sequence information-based features yield similar predictive performance. The combination of two features from different groups performs better than two single features. For example, the accuracy of CTDD and DPC is 0.814 and 0.803 respectively, but their combined accuracy can be improved to 0.818. Therefore, it can be concluded that physicochemical properties-based features and sequence information-based features contribute to the description of amyloidogenic fragments, and they can complement each other to improve the predictive performance of the model. Among all features, the combination of PseAAC and TPC achieves the highest accuracy (0.828), specificity (0.921), Matthew correlation coefficient (0.619), and AUC value (0.890). It may be attributed to the full fusion of amino acid composition, physicochemical properties, correlation, and order information. Accordingly, the combination of PseAAC and TPC is effective and reasonable for constructing predictive models of amyloidogenic regions. Therefore, we adopted a multi-information fusion approach combining PseAAC and TPC to characterize amyloidogenic regions in this study.

#### Validation of feature selection strategy

In our method, 38 PseAAC features and 8000 TPC features were collected from samples in total. The number of features is significantly larger than that of samples, thus we should select the most representative features to reduce the time consumption and overfitting risk. Considering that PseAAC features reflect different physicochemical properties of amyloidogenic fragments and the number of them is sufficiently small, our screening only focuses on the 8000 tripeptide features.

To address the problem, the BD method was exploited to measure the confidence level of every tripeptide feature and the features were arranged in descending order of confidence level. It can be seen that some features showed extremely low confidence levels, even equal to zero. Obviously, these features with low confidence levels were pointless to distinguish amyloidogenic fragments so that they should be removed. Meanwhile, if too many features were removed, the remained features may not enough to describe the amyloidogenic fragments accurately. Upon comprehensive consideration, the threshold of confidence level was set to 0.85. To further improve the feature set, the features with confidence levels around 0.85 were used as cut-off features for constructing corresponding candidate feature sets. Subsequently, these candidate feature sets were fed into a random forest algorithm to predict the amyloidogenic fragments and the feature set consisting of the top 298 tripeptide features was selected as the optimal feature set based on the prediction performance. Finally, the combination of the 38 PseAAC features and the top 298 tripeptide features was used to characterize protein samples in the following sections.

**Fig. 2:**
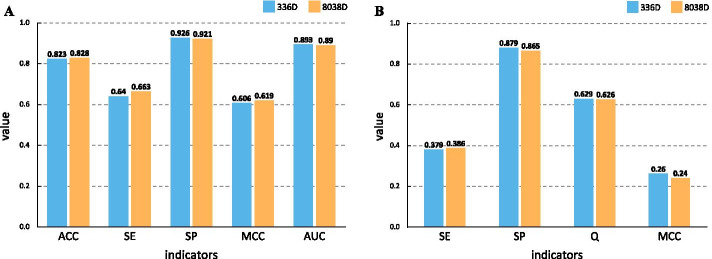
Comparison of prediction performance before and after feature selection. **A** The results on the training set. **B** The results on the Reg33 test set

To verify the effectiveness of the selected features, we performed several comparison experiments. First, we compared the performance differences of amyloidogenic fragments recognition models before and after feature selection on the training set, and the results are shown in Fig. 2A. It can be observed that the model trained with 336 selected features performs as well as the model trained with 8038 original features. This result suggests that the 336 selected features can replace the original features to describe characteristics of amyloidogenic fragments accurately. It may attribute that the 336 selected features are the most representative features of the original features, which can cover the semantics of the original features.

Moreover, high-dimensional features would increase the overfitting risk of machine learning models. To further evaluate the impact of features on the amyloidogenic regions prediction model, the 336 selected features and the 8038 original features were used to predict amyloidogenic regions on the Reg33 test set respectively. The performance of the two models was compared by four metrics: SE, SP, Q, and MCC. In the comparison, as shown in Fig. 2B, the model using the 336 features achieves better performance than the model using the 8038 features in terms of SP, Q, and MCC. The results indicate that the low-dimensional features can effectively reduce the overfitting risk of the model and strengthen the generalization ability of the model.

**Table 2:** Compared time consumption of models using different features

Task	Length	Quantity	T_8038D (s)	T_336D (s)	Time_diff (s)	Improved_rate (%)
1	20	20	7.65	3.2	4.45	58.17
2	20	50	16.81	3.48	13.33	79.30
3	20	100	31.64	5.64	26	82.17
4	40	20	15.61	3.24	12.37	79.24
5	40	50	38.5	6.78	31.72	82.40
6	40	100	72	11.72	60.28	83.72
7	60	20	24.23	4.34	19.89	82.09
8	60	50	57.91	9.47	48.44	83.65
9	60	100	132	22.72	109.28	82.79

Furthermore, to evaluate the effect of features on the running time of the model, the models using different features were applied on multiple amyloidogenic regions prediction tasks and their time consumption was compared. The comparison results are listed in Table 2. “Length” represents the length of the input sequence, “Quantity” represents the number of input sequences, “T_8038D” represents the running time of the model using the 8038 features, “T_336D” represents the running time of model using the 336 features, “Time_diff” represents the time difference between T_8038D and T_336D, and “Improved_rate” represents the improvement rate of the model using the 336 features over the model using the 8038 features in running time. It is obvious that fewer features take less time on the same prediction tasks. Moreover, the greater the predicted workload, the more significant the difference in running time. Thus, the selected features can considerably reduce the running time and improve the efficiency of the amyloidogenic regions prediction model.

Overall, the performance of the model on the 336 selected features is almost the same as the model on 8038 original features. The selected features can effectively reduce overfitting risk and time consumption. Therefore, our feature selection strategy is reasonable and beneficial to predict amyloidogenic regions.

#### Analysis of feature contribution

**Fig. 3:**
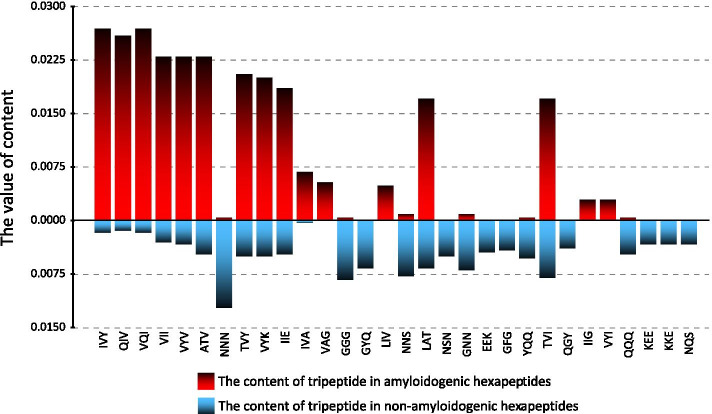
The content of 30 tripeptides with high confidence level

To further reveal the general pattern of tripeptide occurrence in amyloidogenic and non-amyloidogenic fragments, we conducted a statistical analysis. By utilizing the BD method, we sorted the tripeptide features by confidence level and created a ranking list. Figure 3 shows the content of the top 30 tripeptides in the positive and negative samples of the training set, respectively. From Fig. 3, we can discuss the following four aspects. First, the content of each tripeptide differed significantly in positive and negative samples (p-value = 0.018). This suggests that amyloidogenic hexapeptides and non-amyloidogenic hexapeptides have clearly distinguishable tripeptide characterization. Secondly, the content of tripeptides is generally higher in the positive samples compared to the negatives. That is, the occurrence of representative tripeptides in amyloidogenic fragments is more deterministic. Third, the tripeptides with a higher content in the negative samples are basically close or equal to zero in the positives. This indicates that the tripeptide characteristics in the negative sample are more exclusive, and some tripeptides may only exist in non-amyloidogenic fragments. Finally, the amino acids contained in these tripeptides with high confidence levels also present a definite pattern. The predominant Valine and Isoleucine in the positive samples may strongly promote the formation of amyloid fibrils, while the most abundant Asparagine, Glycine, and Glutamine in the negative samples may inhibit the formation of amyloid fibrils. In addition, Valine and Isoleucine are non-polar hydrophobic amino acids, and Asparagine, Glycine, and Glutamine are polar hydrophilic amino acids. Therefore, we can infer that polarity and hydrophobicity are essential to distinguish between amyloidogenic and non-amyloidogenic fragments.

The above results fully illustrate the importance of screening and analyzing tripeptides, and indicate that the selected features are effective for characterizing amyloidogenic fragments.

#### Comparison of different classifiers

In this section, we compared Random Forest with nine well-performing classifiers, including Naïve Bayes, Decision tree, LibSVM, JRip, Multilayer perceptron (MLP), k-Nearest Neighbor (KNN), Locally Weighted Learning (LWL), AdaBoost, and Bagging. In the following, we first give a brief description of them.

The Naive Bayes algorithm is based on Bayesian theory. Its idea is to solve the occurrence probability of the sample to be classified in each category and use it as a basis for classification. The Decision tree is an algorithm for making decisions based on tree structures, which searches decisive features and divides unknown datasets according to the concept of entropy in informatics. Support vector machine was first proposed by Vapnik et al. in 1995. Its idea is to map the feature space to a high-dimensional space and classify data elements by computing the distance from the data points to the separating hyperplane. LibSVM is a software developed by Lin et al. to implement SVM. MLP is a feed-forward artificial neural network that compares the output values with the actual values during training and continuously updates the weights until the prediction error is sufficiently small. JRip, or RIPPER algorithm, is a rule induction learning algorithm with good pruning and stopping principles that remain highly efficient on noisy datasets. Both KNN and LWL are lazy learning algorithms, which means that the model is trained after receiving a test sample. KNN works by finding the k training samples nearest to a given test sample and determining the category of the given sample based on these k “neighbors”, while LWL adds a concept of weighting. These single classifiers have different characteristics and differences. The ensemble learning algorithm integrates the constructed multiple single classifiers according to some strategies to process the learning task. Both Boosting and Bagging are commonly used ensemble classifiers. The individual learners of the Boosting algorithm have strong dependencies and must be generated serially, while the individual learners of the Bagging algorithm do not have strong dependencies and thus can be generated in parallel. The AdaBoost we compared is a representative Boosting algorithm, which can be used for classification and regression.

**Table 3:** Comparison of random forest with other classifiers

	ACC	SE	SP	MCC
Random forest	0.823	0.640	0.926	0.606
AdaBoost	0.751	0.605	0.834	0.450
Bagging	0.796	0.650	0.879	0.549
Naïve Bayes	0.773	0.693	0.818	0.510
LibSVM	0.796	0.585	0.916	0.545
Decision tree	0.779	0.658	0.848	0.515
LWL	0.732	0.581	0.817	0.408
JRip	0.773	0.583	0.880	0.492
KNN (K = 3)	0.791	0.566	0.918	0.532
MLP	0.454	0.734	0.296	0.031

The results of the comparison with the above classifiers are shown in Table 3. We can observe that Random Forest outperformed other classifiers in three metrics of ACC, SP, and MCC. In the SE metric, Random Forest is slightly lower than Bagging, Naïve Bayes, Decision Tree, and MLP by about 0.01-0.094, but higher than them by about 0.047-0.63 in the SP metric. Especially, the specificity of MLP with the highest sensitivity is only 0.296, which verify that it is biased to classify peptides as positives.

**Fig. 4:**
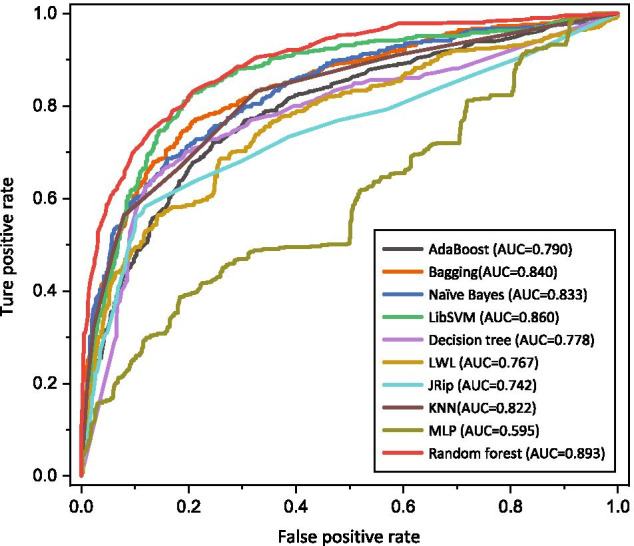
ROC curves for different classifiers

We further used the ROC curve to evaluate the generalization performance of each classification model. From Fig. 4, we could clearly observe that the AUC of Random Forest reaches 0.893, which is significantly better than other classifiers. This demonstrates the superior overall performance and excellent recognition capability of our model.

### Performance of ReRF-Pred in identification of amyloidogenic peptides

After constructing the prediction model on the training set, we compared it with several other state-of-the-art methods. The first was to evaluate the ability of the predictor in distinguishing between amyloidogenic and non-amyloidogenic peptides on the Pep-251 dataset. Based on the assumption that a peptide was predicted as amyloidogenic if at least one amyloidogenic fragment was predicted in it. ReRF-Pred was compared with six methods, AGGRESCAN [12], Waltz [13], MetAmyl [20], PASTA 2.0 [30], APPNN [21], and AmyloGram [22] which provide online servers or software packages and allow multiple sequences to be input simultaneously. PASTA 2.0 used the “peptides” mode suggested by the author, and other methods used default parameters. The results are shown in Table 4. We can see that ReRF-Pred performs best in accuracy (0.801) and Mathew’s correlation coefficient (0.552) except for PASTA 2.0. PASTA 2.0 yielded the best overall performance. Its specificity reaches 0.983, but the sensitivity is only 0.506. This indicates that PASTA 2.0 identifies most amyloidogenic peptides as non-amyloidogenic peptides, and its performance may be limited if other datasets are used.

Collectively, ReRF-Pred can successfully identify amyloidogenic small peptides and achieve a better balance between sensitivity and specificity. It means that our method is feasible for characterizing and predicting hotspots.

**Table 4:** Performance of ReRF-Pred and other methods in peptides identification

	ACC	SE	SP	MCC
ReRF-Pred	0.801	0.734	0.831	0.552
AGGRESCAN	0.741	0.911	0.663	0.534
Waltz	0.765	0.443	0.913	0.414
MetAmyl	0.749	0.924	0.669	0.551
PASTA 2.0	0.833	0.506	0.983	0.603
APPNN	0.769	0.848	0.733	0.542
AmyloGram	0.781	0.823	0.762	0.549

### Performance of ReRF-Pred in prediction of amyloidogenic regions

The purpose of ReRF-Pred is to predict amyloidogenic regions in proteins and reveal their biological characteristics. We evaluated the predictive power of ReRF-Pred on 33 proteins annotated with hotspot regions by comparing it with eight existing methods [12–14, 17, 19–21, 30]. The results are listed in Table 5. The performance of the consensus prediction method AmylPred2 may be weakened if some methods which are base models of the ensemble server cannot work.

**Table 5:** Performance of ReRF-Pred and other existing methods in amyloidogenic regions prediction

	SE	SP	Q	MCC
ReRF-Pred	0.379	0.879	0.629	0.26
Waltz	0.197	0.928	0.562	0.16
AGGRESCAN	0.353	0.792	0.572	0.13
FoldAmyloid	0.275	0.860	0.567	0.13
FISH Amyloid	0.141	0.938	0.540	0.11
MetAmyl	0.525	0.717	0.621	0.19
AmylPred2	0.315	0.894	0.604	0.22
PASTA 2.0 (90% spec)	0.270	0.905	0.588	0.20
PASTA 2.0 (85% spec)	0.381	0.858	0.620	0.23
APPNN	0.537	0.696	0.617	0.18

It is worth noting that the ACC metric is not suitable for the amyloidogenic region prediction tasks. We also take the calcitonin sequence mentioned above as an example, if the hotspot region is predicted to be TYTQDFNKFHTFP, the accuracy is 0.781; however, if all hotspot residues are predicted to be regular residues, the accuracy can reach 0.813. Obviously, the hotspot of the first prediction is better matched, but the accuracy of the first prediction is lower than that of the second one. The reason for this situation is that the number of hotspot residues is usually much smaller than that of regular residues in the amyloidogenic region prediction task. To avoid this situation, a balanced accuracy named Q was introduced in this section, which is the average of sensitivity and specificity scores. For the above example, the values of the Q metric for the two predictions are 0.865 and 0.50, respectively. The second prediction is weaker than the first, which is consistent with common perception.

As we can see from Table 5, ReRF-Pred has the best Q (0.629) and MCC (0.26), which is the most balanced of all methods. Moreover, the MCC of six methods failed to reach 0.20. For PASTA 2.0, which performs best on the Pep-251 dataset, we adjusted its parameter to “90% spec” and “85% spec” for experiments, respectively. The results show that the overall performance of PASTA 2.0 is inferior to that of ReRF-Pred in both experiments. In general, our proposed method allows more efficient detection of amyloidogenic regions in proteins.

The above results based on traditional metrics give us an intuitive performance comparison of the methods. It is worth noting that some methods obtained better overall performance than others but they could not make a precise prediction of amyloidogenic regions on most of the proteins. This was probably because these methods identified more amyloidogenic residues from different proteins than others. Therefore, these methods may have predictive biases for different proteins, which should be taken into account in model evaluation. To solve this problem, we employed the statistical test to further evaluate the performance differences of ReRF-Pred and the concerned methods on each protein.

**Fig. 5:**
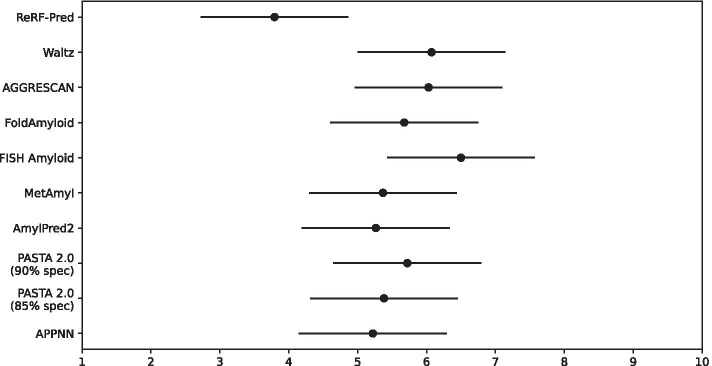
The statistical differences between ten methods

The predictive performance of the ten methods on each protein was ranked from 1 to 10, and the smaller ranking value indicates the superior performance of the method. The Friedman test is widely used for the significance analysis of multiple algorithms in the fields of biology, chemistry, and medicine. Here, the Friedman test with a confidence level of 0.1 was used to determine whether different methods exhibited the same predictive performance. If yes, it suggests that there was no performance difference between the methods. If not, it means that there was a performance difference between methods, and then the Nemenyi post hoc test was utilized to further analyze whether the performance difference between any two methods is significant.

The results of the statistical test are shown in Fig. 5. The x-axis represents the ranking values of the prediction methods, the y-axis represents the names of ten methods, the solid dot represents the average ranking value of the methods on all proteins, and the horizontal line represents the range of Nemenyi’s critical difference. The farther distance between two horizontal lines indicates the more significant performance difference between the two methods. As shown in Fig. 5, ReRF-Pred has the best performance and significantly outperforms Waltz, AGGRESCAN, and FISH Amyloid.

In summary, the effectiveness and robustness of our proposed method can be proved by traditional metrics and statistical tests. In the future, it will greatly promote further studies on the function and mechanism of amyloid.

## Conclusions

Identifying amyloidogenic regions is a basic pathway to find new therapeutic targets for several human complex diseases. In this paper, we proposed a new method for predicting amyloidogenic regions based on sequence information, called ReRF-Pred. The method adopted a multi-feature encoding strategy to combine pseudo amino acid composition and tripeptide composition of amino acids to characterize hotspots of proteins accurately. According to experimental results, our novel approach can achieve an accuracy of 0.823 on the training set through 10-fold cross-validation. What is more, when performed on two independent validation datasets, our method still displayed promised performance. For example, when conducted on the Reg33 dataset, it is superior to the concerned methods for predicting hotspot regions in terms of two important metrics: Q and MCC, which reached up to 0.629 and 0.26 respectively. It is suggested that PseAAC and TPC are effective features to characterize hotspots of amyloidosis. Furthermore, it can be concluded that polarity and hydrophobicity play crucial roles during the process of amyloidosis by further analyzing tripeptides that are significantly distributed differently between positive and negative sample sets. We also provided a web server that allows multiple sequences to be predicted simultaneously, which is available from http://106.12.83.135:8080/ReRF-Pred/.

## Supplementary information

**Additional file 1.** The three datasets used in this paper: Training dataset, Pep-251, and Reg33.

## Data Availability

The datasets used during the present study are available from Additional Files.

## Abbreviations

PseAAC: Pseudo amino acid composition
TPC: Tripeptide composition
SVM: Support vector machine
PSSM: Position-specific scoring matrix
BD: Binomial distribution
CL: Confidence level
ACC: Accuracy
SE: Sensitivity
SP: Specificity
MCC: Mathew’s correlation coefficient
ROC: Receiver operating characteristic
AUC: Area under curves
CTriad: Conjoint triad
AAC: Amino acid composition
DDE: Dipeptide deviation from expected mean
DPC: Dipeptide composition
MLP: Multilayer perceptron
KNN: k-Nearest neighbor
LWL: Locally weighted learning
