# Supplementary material for: ReRF-Pred: predicting amyloidogenic regions of proteins based on their pseudo amino acid composition and tripeptide composition
Source: BMC Bioinformatics. 2021 Nov 9;22:545. doi: 10.1186/s12859-021-04446-4 (PMC8579573; doi:10.1186/s12859-021-04446-4)
Supplement: Supplementary file 1 — Additional file 1. The three datasets used in this paper: Training dataset, Pep-251, and Reg33. [file 12859_2021_4446_MOESM1_ESM.docx]

**1.Training dataset**

**Positive samples (511):**

| YYQNYQ | VQIVYG | VAIVYK | SSNNFG | NHFCIQ | LATVYI | IVATTR |
| --- | --- | --- | --- | --- | --- | --- |
| YVEYIG | VQIVYF | VAFNIT | SQVIIE | NFVNYS | LATVYF | ITVIIE |
| YTVIIE | VQIVYE | TYVEYI | SQIVYK | NFLVHS | LATVYE | ITLFWG |
| YQLIWQ | VQIVYD | TTVIIE | SQFYIT | NFGSVQ | LATVYC | ISFLIF |
| YQIVYK | VQIVYC | TQIVYK | SQAIIH | NFGEFS | LATVWV | IQIVYK |
| YNNYNN | VQIVYA | TIITVT | SNVIIE | NFGAIL | LATVVV | IQIMIW |
| YLVNFT | VQIVWK | TIITLE | SNQNNF | NATVYV | LATVTV | ILVAGS |
| YLNWYQ | VQIVVK | TGELAT | SMVLFS | MVGGVV | LATVSV | ILVAGD |
| YLLYYT | VQIVTK | TFQINS | SMVIIE | MTVIIE | LATVRV | ILQINS |
| YLLWYC | VQIVSK | TCVTHR | SLVIIE | MTFIQN | LATVQV | IINFIT |
| YLFLQY | VQIVRK | TCMLGT | SIVIIE | MQIVYK | LATVPV | IIHFGS |
| YIFNNC | VQIVQK | TAVVTN | SGVIIE | MMHFGN | LATVNV | IFQINS |
| YHLAMA | VQIVPK | TAVTHR | SFVIIE | MLVLFV | LATVMV | IFDFIQ |
| YATVYV | VQIVNK | TATVYV | SFQIYA | MIHFGN | LATVLV | IEMIFV |
| YAELIV | VQIVMK | TASNSS | SFLIFL | MIENIQ | LATVKV | IATVYV |
| WVFWIG | VQIVLK | TAMLGT | SFFFIQ | MHVNIQ | LATVIV | HYFNIF |
| WTVNYS | VQIVKK | TAMIIQ | SEVIIE | MCSIFQ | LATVHV | HTVIIE |
| WTVIIE | VQIVIK | SYVIIE | SELNIY | MATVYV | LATVGV | HTFNLL |
| WTFNLY | VQIVHK | SYSTMS | SDVIIE | LYVLIV | LATVFV | HSSNNF |
| WQIVYK | VQIVGK | SVVIIE | SAVIIE | LYTVYV | LATVEV | HQLIIM |
| WPNVIT | VQIVFK | SVSSSY | SATVYV | LYQLEN | LATVDV | HQFNLN |
| WIVIFF | VQIVEK | SVNLDV | SALALQ | LWTVYV | LATVCV | HLVYIM |
| WATVYV | VQIVDK | STWLIE | RVFNIM | LVTVYV | LATVAV | HLFNLT |
| VYIVYK | VQIVCK | STWIYE | RTVIIE | LVHSSN | LATMYV | HIFLLC |
| VYIMIG | VQIVAK | STWIIE | RQVLIF | LVFYQQ | LATLYV | HICNFF |
| VWIVYK | VQITYK | STWIFE | RATVYV | LVEALY | LATIYV | HGWLIM |
| VVSTTV | VQISYK | STVYIE | QYGGNN | LTVIIE | LATFYV | HGFNQQ |
| VVIVYK | VQIRYK | STVWIE | QYGGNA | LTTVYV | LATCYV | HFVWIA |
| VVFFIC | VQIQYK | STVTIE | QYFEQF | LSTVYV | LASVYV | HATVYV |
| VTVQAT | VQINYK | STVNIE | QTVIIE | LSFSKD | LAQVYV | GYVLGS |
| VTVIIE | VQIMYK | STVLIE | QTFLVN | LQTVYV | LANVYV | GYVIIK |
| VTSTFS | VQILYK | STVIYE | QQSLFQ | LQIVYK | LANFLV | GYMLGS |
| VTQVGF | VQIKYK | STVIIY | QQIVYK | LPTVYV | LAMVYV | GYFLNM |
| VTLWWG | VQIIYK | STVIIW | QNLLWQ | LNTVYV | LALVYV | GYFLNF |
| VTLHAT | VQIINK | STVIIV | QLFLQI | LNIYQY | LAIVYV | GYFILQ |
| VTIVYK | VQIFYK | STVIIT | QLFINF | LMTVYV | LAHVYV | GYCFIL |
| VTFTIQ | VQIDYK | STVIIS | QLENYC | LLYYTE | LAGVYV | GVWWFF |
| VSTTVV | VQICYK | STVIIQ | QILLWS | LLTVYV | LAFVYV | GVVTSE |
| VSIVYK | VQFVYK | STVIIN | QGVCFR | LLAVVA | LACVYV | GVQVGD |
| VSFLSA | VQFLQS | STVIIM | QFFLQF | LKVKVL | LAAVYV | GVNYFL |
| VSFEIV | VQCVYK | STVIIL | QEMRHF | LIVAGT | KVQIIN | GVIWIA |
| VRIVYK | VNVTQV | STVIII | QATVYV | LIVAGS | KTVIIE | GVIGIA |
| VQVVYK | VNQLYQ | STVIIG | QANKHI | LIVAGK | KTVEGA | GVATVA |
| VQTVYK | VNIVYK | STVIIF | NYVWIV | LIVAGE | KTKQGV | GTVWWG |
| VQQVYK | VMIVYK | STVIIE | NYCNFV | LIVAGD | KTKEQV | GTVLFM |
| VQNVYK | VLVEAQ | STVIID | NTVIIE | LIVAAD | KTKEGV | GTVIIE |
| VQMVYK | VLIVYK | STVIIA | NQQNQY | LITVYV | KQIVYK | GTLVFM |
| VQLVYK | VKIVYK | STVIFE | NQNNFV | LIFLIV | KQIGII | GTLFWG |
| VQIYYK | VIVATT | STVFIE | NQIVYK | LHTVYV | KNFNYN | GTFNII |
| VQIWYK | VILLIS | STVEIE | NQFNLM | LGTVYV | KNEEGA | GTFFIT |
| VQIVYY | VIIVYK | STSIIE | NQFIIS | LFTVYV | KLVFFA | GTFFIN |
| VQIVYW | VIGIAQ | STQIIE | NNSGPN | LEFWLQ | KLLIYE | GSIAAT |
| VQIVYV | VIFTIM | STNIIE | NNQQNY | LCTVYV | KLFIQF | GNVCIN |
| VQIVYT | VHIVYK | STMSIT | NNQNTF | LAYVYV | KLFIIQ | GNVAVA |
| VQIVYS | VGIVYK | STLIIE | NNNWSL | LAWVYV | KIVKWD | GNTCVN |
| VQIVYR | VFIVYK | STFTFE | NLFYQL | LAVVYV | KHIIVA | GNFNNL |
| VQIVYQ | VFFNNT | STFLIE | NKGAII | LAVLFL | KDWSFY | GNCFIL |
| VQIVYP | VEIVYK | STFIIE | NINKSN | LAVAGD | KCWCFT | GMMGML |
| VQIVYN | VEALYL | STFIFE | NINFIT | LATYYV | KCLNYL | GLSIFV |
| VQIVYM | VDIVYK | STEIIE | NINFFT | LATVYY | KATVYV | GLFINM |
| VQIVYL | VCIVYK | SSVIIE | NIFWVQ | LATVYW | KAKEGV | GLCYFN |
| VQIVYK | VAVHVF | SSTSAA | NIFNIT | LATVYV | IYQYGG | GIVIVA |
| VQIVYI | VATVYV | SSTNVG | NIFNFT | LATVYN | IVYSWE | GIFNIK |
| VQIVYH | VANFIT | SSQVTQ | NHVTLS | LATVYM | IVYSCE | GGYVLG |
| GGYMLG | GATVYV | FIVNIV | DIDLHL | CGVIGI | AIVAGD | DQIVYK |
| GGYLLG | GAITIG | FESNFN | DHCIIW | CATVYV | AILSST | CILFWG |
| GGVVIA | GAILSS | FATVYV | DHCIIG | AVFIIY | AIIGLM | AIVAGS |
| GGVLVN | GAIIGL | FAIRHF | DFNKFH | ATVQAF | AGVNYF | GDHCII |
| GGGNNS | FYLLYY | EYSNFS | DCVNIT | ATVIIE | AEVVFT | FQIVYK |
| GFFQQQ | FVFYIF | ETVIIE | CTVWWG | ASSSNY | AATVYV | DSVISL |
| GEWTYD | FTVIIE | EQIVYK | CTVFIG | AQFIIS | AAIIGQ | CQIVYK |
| GELAVA | FTFIQF | EGVLYV | CTLWWG | ANFLVH | IIGLMV | AIVAGT |
| GDVIEV | FSINPA | DTVIIE | CTIYWG | ALEEYT | GDCFIL | FLVHSS |

**Negative samples (903):**

| AAAQAA | FNPQGG | IHKAQN | LTQRGF | PKVEDL | SGDHCI | TNELYM |
| --- | --- | --- | --- | --- | --- | --- |
| AAELRN | FNYNNN | IINFEQ | LVSSSG | PMINLY | SGFGNS | TNNNTN |
| AAIDWF | FQKQQK | IIPFEQ | MAAAQA | PNGITL | SGFHPS | TQHGGG |
| AAIGWG | FQPQSQ | IITVTN | MERLRI | PNSANS | SGIKLA | TQRGFG |
| AALQSS | FSKDWS | IKLANA | MEWLRI | PNVITL | SGSGFN | TQVGFG |
| AAPKPK | FSNATN | ILENIS | MGGGMN | PQGGRG | SGSNSG | TREPTK |
| AAQAAL | FTPTEK | ILGSNN | MGMLAS | PQGGYQ | SGYQQG | TRNNTV |
| AARRFF | FVNQHL | INLYTD | MGNNTV | PQIVYK | SHLVEA | TTGVTI |
| AAVDQT | FYTPKT | INNMTN | MINLYT | PQSQGM | SHVIIE | TTGVVV |
| ACGVIG | GAAIGW | INNVSN | MLASQQ | PQYGGG | SIDLTQ | TTVVST |
| ADVGQG | GAAVDQ | INPAMM | MLASRQ | PSDIEV | SIEDSV | TTVVTT |
| ADVSIE | GADVGQ | IQNNSN | MMAAAQ | PSGNNQ | SINPAM | TVFHIG |
| AEKLFD | GAFSIN | IQRTPK | MMGMLA | PTEKDE | SKVIIE | TVFPIG |
| AEMEYL | GEATVS | IQVYSR | MNFGAF | PTKVEE | SLNDFQ | TVIVIR |
| AENGKS | GERGFF | ISLSGD | MNFGTF | PTRCCP | SLSGDH | TVKQFG |
| AESDKK | GFFNLQ | ISMTTS | MSDSNQ | PTREPT | SLYQLE | TVVSTT |
| AETKEP | GFFYTP | ITQHGG | MSLNDF | PTVIIE | SNAGSG | TYQIIR |
| AEVLAL | GFGNNA | ITVTND | MTVKQF | PVKKEE | SNGIVI | VAMHVF |
| AFSINP | GFGNQS | IVIRTP | MYFFIF | PVQTEE | SNMGGG | VANNST |
| AGFQPQ | GFGNSA | IVIVAT | MYWIIF | PYGQQS | SNNFGA | VATTRT |
| AGGYYQ | GFGNSR | IYFNVQ | NAGSGS | QAALQS | SNNSNI | VAWLKM |
| AGSGSG | GFNKFG | KAAIDW | NANASS | QAAPKP | SNQGNN | VDQTAS |
| AGYQQQ | GFNNSA | KAFIIQ | NANTSN | QAGFQP | SNSGAA | VDTKPA |
| AIDWFD | GFNNTP | KAILFL | NAQAQP | QAQNQW | SNTNNT | VEDLKI |
| AIGWGS | GFPGSF | KECLIN | NATAHQ | QAQPAG | SPVIIE | VEEPVK |
| AINKIQ | GFQPQS | KEEEKS | NATKKV | QAYNAQ | SQGMSL | VFFINQ |
| AISNNN | GFSGSF | KEEKPV | NDFQKQ | QFGGGN | SQNGNQ | VGFGNN |
| ALALQT | GFSNNN | KEEKSE | NDNNNN | QFNPQG | SQQNQS | VGQGSD |
| ALQSSW | GGFGNQ | KENIIF | NDNNSN | QGFNNQ | SRHPAE | VHNNSN |
| ALQTDA | GGFNKF | KEPTRE | NENGNA | QGGRGN | SRQNQS | VISLSG |
| AMMAAA | GGGGNH | KGENFT | NFFIQS | QGGYQQ | SRVIIE | VIVIRT |
| AMNITN | GGGGNN | KKEEEK | NFFWLL | QGMSLN | SSGIKL | VIWIAQ |
| ANATKK | GGGMNF | KKEEKP | NFGAFS | QGNNQQ | SSIDLT | VKKEEK |
| ANNNSN | GGGNGA | KKEEKS | NFGTFS | QGNNRY | SSNNNS | VKQFGG |
| ANNYSS | GGGNHG | KKTLKL | NFNNGN | QGYQAG | SSSGIK | VKWDRD |
| APKPKK | GGGNSA | KKVDTK | NFNYNN | QGYQAY | SSVNVT | VNNNNI |
| AQAALQ | GGMNFG | KLANAT | NGAAVD | QGYSGY | SSWGMM | VNNNNN |
| AQAQPA | GGNGAA | KLLEIA | NGADVG | QHGGGN | SSYSSY | VNWCER |
| AQIVYK | GGNGAD | KLVSSS | NGERIE | QIGIIK | STADWE | VNWYER |
| AQPAGG | GGNHGG | KMFFIQ | NGIVIV | QKQAAP | STAIIE | VPGIAQ |
| ASNAGS | GGNNSG | KNFNYN | NGKSNF | QKQQKQ | STDIIE | VPIVYK |
| ASQQNQ | GGNSAL | KPAESD | NGNLSN | QLFINN | STFTIE | VPQYGG |
| ASRQNQ | GGQQQS | KPKKTL | NGNLTS | QNGNQQ | STGIIE | VQAVYK |
| ATAHQY | GGRGNY | KPVQTE | NGNNSN | QNQSCP | STHIIE | VQDVYK |
| ATKKVD | GGYQQQ | KQAAPK | NGNQNR | QNQSGP | STIIIE | VQEVYK |
| ATLDQW | GGYQQY | KQFGGG | NGNQQQ | QNQYNS | STKIIE | VQGVYK |
| ATTRTV | GGYYQN | KQQKQA | NHGGGG | QNSLNM | STLIFE | VQHVYK |
| ATVSFD | GIIKTN | KSAETK | NIDQSF | QNYQGY | STLIYE | VQIAYK |
| AVDQTA | GIKLAN | KSELPK | NIGNNS | QNYQQY | STLLIE | VQIEYK |
| AVTTGV | GKQAYQ | KSNFLN | NIGQSF | QPAGGY | STLLYE | VQIGYK |
| AYNAQA | GKQGYQ | KTLKLV | NININV | QPKIVK | STLTFE | VQIHYK |
| CPSGNN | GLLLWQ | KTVIIT | NINNNN | QPMINL | STLTIE | VQIPYK |
| DADLYL | GMFNIQ | KTVIVE | NINNSI | QPQSQG | STMIIE | VQKVYK |
| DAGYQQ | GMLASQ | KTVIYE | NINNST | QQFNPQ | STNIFE | VQPVYK |
| DATVYV | GMLASR | KTVLIE | NIQYQF | QQGGYQ | STNIYE | VQRVYK |
| DDSLFF | GMNFGA | KVDTKP | NISNNN | QQGNNR | STNNTN | VQSVYK |
| DECFFF | GMNFGT | KVEDLK | NIVLIM | QQKQAA | STNSNT | VQTEEK |
| DENNTF | GMSLND | KVEEPV | NIYQYG | QQNQSC | STNTFE | VQWVYK |
| DETVIV | GNGAAV | KVEHSD | NKFGGP | QQNQSG | STNTIE | VQYVYK |
| DFQKQQ | GNGADV | KWDRDM | NLGPVL | QQNQYN | STPIIE | VSIEDS |
| DIEVDL | GNHGGG | KYLNWD | NLQGYQ | QQNTSF | STQIFE | VSNLSN |
| DILTYT | GNNATA | KYNNTG | NLYTDR | QQNYQQ | STQIYE | VSSSGI |
| DKKEEE | GNNQQN | LADVYV | NMGGGM | QQNYSK | STRIIE | VTIITV |
| DLLKNG | GNNRYQ | LAEAIG | NMNMNL | QQQFNP | STTIIE | VTLSQP |
| DLTITQ | GNNSGP | LAEVYV | NNATAH | QQQGNN | STTVVT | VTQEFW |
| DLTQRG | GNNSYS | LAKVYV | NNGGNN | QQQSYG | STVAIE | VTTGVT |
| DNNTIF | GNQQQG | LALQTD | NNLQGY | QQQSYN | STVDIE | VTTGVV |
| DQTASN | GNQSGF | LANATK | NNNGNQ | QQQYNP | STVGIE | VTTVVS |
| DQWNGK | GNSALA | LAPVYV | NNNISN | QQSYGQ | STVHIE | VVPQYG |
| DSNQGN | GNSATL | LARVYV | NNNISV | QQSYNP | STVIAE | VVTSEE |
| DTKPAE | GNYKNF | LASQQN | NNNKNN | QQSYSG | STVIDE | VVVTSE |
| DVGQGS | GQGSDD | LASRQN | NNNLQG | QQYNPD | STVIEE | VVVVTS |
| DVNRNG | GQIVYK | LATAYV | NNNNNE | QQYNPQ | STVIGE | VYSCEW |
| DVSIED | GQNNTQ | LATDYV | NNNNSN | QQYSQN | STVIHE | VYSRHP |
| EALYLV | GQQNQY | LATEYV | NNNNTN | QRGFGN | STVIIH | VYSWEW |
| EATVSF | GQQQSY | LATGYV | NNNRGG | QSCPSG | STVIIK | WGMMGM |
| EATVYV | GQQSYS | LATHYV | NNNSSS | QSGFGN | STVIIP | WGSASN |
| ECFFFE | GQSSYS | LATKYV | NNQQNY | QSQGMS | STVIIR | WNFGTQ |
| EDSVIS | GRGNYK | LATNYV | NNRYQG | QSSWGM | STVIKE | WNFSTQ |
| EECLFL | GRIGIA | LATPYV | NNSYSG | QSSYSS | STVIKT | WPNGIT |
| EEEKSA | GSASNA | LATQYV | NNTIFV | QSYGQQ | STVILE | WSFYLL |
| EEKKEE | GSGFNG | LATRYV | NPAMMA | QSYNPP | STVIME | WVENYP |
| EEKPVQ | GSGSGF | LATSYV | NPDAGY | QSYSGY | STVINE | WYFYIQ |
| EEKSAE | GSHLVE | LATTYV | NPQGGR | QTDARN | STVIPE | YASEIE |
| EEKSEL | GSNNTF | LATVYA | NPQGGY | QTEEKK | STVIQE | YASNNN |
| EEPVKK | GSNSGA | LATVYD | NQGNNQ | QTFNLF | STVIRE | YGGGGN |
| EHSDLS | GTFFQE | LATVYG | NQGYNT | QTNLYG | STVISE | YGGGNS |
| EKDEYA | GTFSIN | LATVYH | NQNMNN | QVGFGN | STVITE | YGQQNQ |
| EKKEEK | GVFNNQ | LATVYK | NQQNYQ | QVVHIG | STVIVE | YGQQQS |
| EKNLYL | GVNNNN | LATVYL | NQQQGN | QVVPIG | STVIWE | YGQQSY |
| EKPVQT | GVPGIA | LATVYP | NQSCPS | QWNGKN | STVKIE | YGQSSY |
| EKSAET | GVTIIT | LATVYQ | NQSGFG | QYGGGG | STVMIE | YIFNNM |
| EKSELP | GVVPQY | LATVYR | NQYNSS | QYGGGN | STVPIE | YKNFNY |
| ELNIYQ | GVVVVT | LATVYS | NRYQGY | QYNNSN | STVQIE | YLEIII |
| ELPKVE | GWGSAS | LATVYT | NSALAL | QYNPDA | STVRIE | YLGNNS |
| EMTVKQ | GYGQSS | LATWYV | NSATLD | QYNPQG | STVSIE | YNAQAQ |
| EPTKVE | GYQAGF | LDGFSR | NSGAAI | QYSQNG | STVTFE | YNNNLQ |
| EPTREP | GYQAYN | LDGSSR | NSNINQ | REPTKV | STVVIE | YNNTGS |
| EPVKKE | GYQQGG | LDQWNG | NSNLTA | RETWFF | STWTFE | YNPDAG |
| ERGFFY | GYQQQF | LDTVYV | NSNNSN | RGFFYT | STWTIE | YNPQGG |
| ERIEKV | GYQQQY | LETVYV | NSNSSN | RGFGNS | STYIIE | YQAGFQ |
| ESDKKE | GYQQYN | LFMFND | NSNTNT | RGGFNK | SVISLS | YQAYNA |
| ESNTNN | GYSGYQ | LFMSND | NSNTTK | RGNYKN | SVNVTQ | YQGYQA |
| ETKEPT | GYYQNY | LFNNST | NSNYTQ | RIGIAQ | SWGMMG | YQGYSG |
| ETVIVI | HAFLII | LGNNSN | NSSFNG | RLSNNI | SWVIIE | YQLENY |
| ETWFFG | HGGGGN | LIAGFN | NSYSGS | RLVFID | SYLCDL | YQNYQG |
| EVDLLK | HGGGNG | LINNSV | NTIFVQ | RMFNII | SYLYDL | YQQGGY |
| EVTTGV | HIFIIM | LKLVSS | NTNNTS | RNLTKD | SYNNNN | YQQQFN |
| EYLKIA | HKALFW | LKNGER | NVAMHV | RQFNQT | SYSGSN | YQQQYN |
| FERQHM | HLVEAL | LKTVYV | NVAVHV | RQIVYK | TAAHCF | YQQYNP |
| FFNLNN | HNNNNN | LLFCHI | NVNFSN | RQNQSG | TAELIT | YQQYSQ |
| FFWRFM | HNNNSQ | LLFYHI | NVTQVG | RTPKIQ | TAVHCF | YQYGGG |
| FFYTPK | HNSVNF | LLGPTI | NYFAIR | RVAFFE | TAVTTG | YSGSNS |
| FGAFSI | HNTNNA | LLNKTN | NYKNFN | RVNHVT | TAWYAE | YSGYQQ |
| FGELFE | HPAENG | LLVPTI | NYNNNL | RVNNNA | TDETVI | YSQNGN |
| FGGGNG | HQIVYK | LMNTTN | NYNNSG | RYQGYQ | TEEKKE | YTFTIS |
| FGNNAT | HTAVTT | LMSLFG | NYNNSS | SAETKE | TEFTPT | YTNISN |
| FGNQSG | HWQNFA | LNDFQK | NYNTYR | SASNAG | TFSINP | YVSGFH |
| FGNSAT | HWRNFA | LNNITA | NYQGYS | SATLDQ | TFWEIS | YYNYNN |
| FGNSRG | ICSLYQ | LPKVED | NYQQYS | SCNNNN | TGVTII | YYQNYQ |
| FGTFSI | IDLTQR | LQGYQA | PAESDK | SCPSGN | TGVVVV | YYTEFT |
| FHPSDI | IEDSVI | LQNVSN | PAGGYY | SDKKEE | TITQHG | KSHPET |
| FINYTN | IEKVEH | LQSSWG | PAMMAA | SDLSFS | TKEPTR | SGDGSL |
| FKQIGI | IFNYNN | LQTDAR | PATVYV | SDLTIT | TKKVDT | EIPAGV |
| FLKYFT | IGIIKT | LRTVYV | PDAGYQ | SDNNTI | TKPAES | DAVKIS |
| FMFFII | IGNNSI | LSGDHC | PGGFGN | SDSNQG | TKVEEP | ETYVVT |
| FMFNDL | IGRTLV | LSQPKI | PKIQVY | SELPKV | TLDQWN | TYSFYC |
| FMSNDL | IGRTLW | LTFIQV | PKKTLK | SEMTVK | TLKLVS | NKGAIK |
| FNKFGG | IGWGSA | LTITQH | PKPKKT | SGAAIG | TLKNYI | VTKVAK |

**2. Pep-251 dataset**

| class | seq |
| --- | --- |
| + | PGGGKVQIVYKPV |
| + | NLKHQPGGGKVQIVYKEVD |
| + | GKVQIVYK |
| + | DAEFRHDSGYEVHHQKLVFFAEDVGSNKGAIIGLMVGGVV |
| + | VPHQKLVFFAEDVGS |
| + | VHPQKLVFFAEDVGS |
| + | VHHPKLVFFAEDVGS |
| + | VHHQPLVFFAEDVGS |
| + | VHHQEKLVFFAEPVGS |
| + | VHHQEKLVFFAEDPGS |
| + | VHHQEKLVFFAEDVPS |
| + | KKLVFFAED |
| + | VHHQKLVFFAEDVGS |
| + | KKLVFFPED |
| + | HHQKLVFFAED |
| + | VHHQKLVFFAEDV |
| + | EVHHQKLVFFAEDVG |
| + | YEVHHQKLVFFAEDVGS |
| + | GYEVHHQKLVFFAEDVGSN |
| + | SGYEVHHQKLVFFAEDVGSNK |
| + | DSGYEVHHQKLVFFAEDVGSNKG |
| + | HDSGYEVHHQKLVFFAEDVGSNKGA |
| + | EQVTNVGGAVVTGVTAVA |
| + | TVNGVGEVTATAVQGVAV |
| + | VTNVGGAVVTGVTAVA |
| + | EQVTNVGGAVVTG |
| + | VGGAVVTGV |
| + | GVVGWVKNTSKGTVTGQVQG |
| + | ISKLEYSNFSVRY |
| + | NLKHQPGGGKVQIVYKPVDLSKVTSKCGSLGNIHHKPGGGQVE |
| + | DWSFYLLYYTEFT |
| + | DWSFYLLYYTEFTPTGKDEYA |
| + | TKRPRFLYEIAMALNSD |
| + | VLSEGEWQLVLHVWAKVEA |
| + | EGEWQLVLHVWAKVEADVAGHGQDILIRLFK |
| + | DVAGHGQDILIRLFKS |
| + | EVVPHKKMHKDFLEKIGGL |
| + | SAPNLATLVKVTTNHFTHEEAMMD |
| + | LEVLLGSGDGSLVFV |
| + | IPAGVDAVKISM |
| + | GETYVVTL |
| + | DILTLLNSTNKDWWKVEVND |
| + | GEKIVFKNNAGFPHNVVFDE |
| + | MPEEELLNAPGETYVVTL |
| + | MKVIFLKDVKG |
| + | GTYSFYT |
| + | LSQTFVYGGSRAKRNN |
| + | GYANNFLFKQG |
| + | GTVSFVTSPHQGAGMVGKVTVN |
| + | QISFADYNLLDLLRIHQVLN |
| + | DWWKVEVNDRQGFVPA |
| + | FVNVQAVKVFLESQGIAY |
| + | FVNVEAVKAFLEAHGIAY |
| + | STNVKTAFEMVILDIYNNV |
| + | TESKEKITQYIYHVLNGEIL |
| + | AKKENIIAAAQAGASGY |
| + | PFTAATLEEKLNKIFEKLGMY |
| + | GVGKSALTIQLIQNHFVY |
| + | RQGVEDAFYTLVREIRQHK |
| + | VTIKANLIFANGFTQTAEFKG |
| + | KGTFEKATSEAYAYADTLKKDNGEY |
| + | GEYTVDVADKGYTLNIKFAGD |
| + | GEWTYDDATKTFTVTE |
| + | VHDCVNITIK |
| + | SMVLFSSPPV |
| + | SSPPVILLIS |
| + | ILLISFLIFL |
| + | FLIFLIVG |
| + | RCELARTLKR |
| + | LANWMCLAKW |
| + | DLSFSKDWSF |
| + | KDWSFYLLYY |
| + | YLLYYTEFTP |
| + | TEFTPTEKDE |
| + | ETLKMSMFLEAQFKKSAL |
| + | ASSQKKMKEMLAFFTLEL |
| + | KLELKAASQMEFSFTMKL |
| + | KELKQELFFKASATLMMS |
| + | SAFMEKMLLLEKQFKAST |
| - | PGGGKVYKPV |
| - | PGGGKNAEVYKPV |
| - | PGGGKVQIVEKPV |
| - | QTAPVPMPDLKNVKSKIGSTENLKHQPGGGKVQIVY |
| - | KPVDLSKVTSKCGSLGNIHHKPGGGQVEVKSEKLDF |
| - | KDRVQSKIGSLDNITHVPGGGN |
| - | QTAPVPMPDLKNVKSKIGSTE |
| - | KLDFKDRVQSKIGSLDNITHVPGGGN |
| - | QTAPVPMPD |
| - | LKNVKSKIGSTE |
| - | LSKVTSKCGSLGNIHHKPGGGQVE |
| - | VKSEKLDFKDRVQSKIGSLDNITHVPGGGN |
| - | VDLSKVTSK |
| - | VTSKCGSLGNIHHKPGGG |
| - | GQVEVSKE |
| - | KKPVFFAED |
| - | KKLPFFAED |
| - | KKLVPFAED |
| - | VHHQKLVPFAEDVGS |
| - | KKLVFPAED |
| - | VHHQEKLVFFAPDVGS |
| - | QKLVFFA |
| - | HQKLVFFAE |
| - | STAQSLKSVDYEVFGRV |
| - | QGVSFRMYTEDEARKI |
| - | PEDKVNSMKSWLSKV |
| - | GSPSSRIDRTNFSNEKT |
| - | IQRTPKIQVYSRHPAE |
| - | NGKSNFLNCYVSG |
| - | FHPSDIEVDLLK |
| - | NGERIEKVEHSDLSFSKD |
| - | PTGKDEYACRVNHVT |
| - | LSQPKIVKWDRDM |
| - | MQTLSERLKKRRIALKY |
| - | YKMTQTELATKAGVK |
| - | YKQQSIQLIEAGVTKR |
| - | AMALNCDPVWLQYGTKRGKA |
| - | HPETLEKFDRFKHLK |
| - | TEAEMKA |
| - | SEDLKKHGVTVLTALGAILK |
| - | KKGHHEAE |
| - | ELKPLAQSHA |
| - | ATKHKIP |
| - | GWEIPEPYVWDESFRVFY |
| - | GTDFKYKGKL |
| - | YEQLDEEHKKIFKGIFDCIRD |
| - | DAAKYSEV |
| - | GLSAPVD |
| - | AKNVDYCKEWLVNHIK |
| - | LEVLLGSG |
| - | SLVFVPSEFS |
| - | SEFSVPSGEK |
| - | KIVFKNNA |
| - | KIVFKNNAGFPH |
| - | KNNAGFPHNV |
| - | PHNVVFDEDDEIP |
| - | MPEEELL |
| - | ELLNAPGETY |
| - | NAPGETY |
| - | VTLDTKGTY |
| - | YTSPHQGAGMV |
| - | MVGKVTVN |
| - | RPDFSLEPPYTGPSK |
| - | PSKARIIRY |
| - | KRNNFKSAEDS |
| - | ARIIRYFYNAKAG |
| - | FKSAEDSMRTSGGA |
| - | NAKAGLSQT |
| - | KGKKGEIKNVAD |
| - | LAIEATPA |
| - | TPANLKALEAQKQKEQR |
| - | DQKEAALVDMVNDGVEDLRCKYATLIYT |
| - | YEAGKEKYVKELPEHLKPFETLLSQ |
| - | PLLSAYVARLSA |
| - | PKIKAFLA |
| - | AYVKKLDSGTGKELVLAL |
| - | YDYQEKSPREVTMKKGD |
| - | GGKDWWKVGG |
| - | DILTLLNSTNKDWWKVEVNDRQGFVPA |
| - | VPSNEEQIKNLLQLEAQEHLQY |
| - | VPSNEEQIKKLLELEAKKHLQY |
| - | AVGKSNLLSRYARNEFSA |
| - | RFRAVTSAYYRGAVG |
| - | TRRTTFESVGRWLDELKIHSD |
| - | AVSVEEGKALAEEEGLF |
| - | DHPAVMEGTKTILETDSNLS |
| - | EPSEQFIKQHDFSSY |
| - | VNGMELSKQILQENPH |
| - | EVEDYFEEAIRAGLH |
| - | DFSTMRRIVRNLLKELGYN |
| - | EDGVDALNKLQAGGY |
| - | MDGLELLKTIRADSAY |
| - | GTGNTEKMAELIAKGIIESGKDY |
| - | EESEFEPFIEEISTKISY |
| - | GDGKWMRDFEQRMNGYGSV |
| - | EPDEAEQDSIEFGKKIANIY |
| - | EYSAMRDQYMRTGEG |
| - | INNTKSFEDIHQYREQIKRVKDS |
| - | ARTVESRQAQDLARSYGIP |
| - | TYKLINGKTLKGETTTEA |
| - | GDAATAEKVFKQYANDNGVD |
| - | QGGGTHSQWN |
| - | HSQWNKPSKP |
| - | KPSKPKTNMK |
| - | KTNMKHMAGA |
| - | HMAGAAAAGA |
| - | AAAGAVVGGL |
| - | VVGGLGGYML |
| - | GGYMLGSAMS |
| - | GSAMSRPIIH |
| - | FGSDYEDRYY |
| - | EDRYYRENMH |
| - | RENMHRYPNQ |
| - | RYPNQVYYRP |
| - | VYYRPMDEYS |
| - | MDEYSNQNNF |
| - | NQNNFVHDCV |
| - | NITIKQHTVT |
| - | QHTVTTTTKG |
| - | TTTKGENFTE |
| - | ENFTETDVKM |
| - | TDVKMMERVV |
| - | MERVVEQMCI |
| - | EQMCITQYER |
| - | TQYERESQAY |
| - | ESQAYYQRGS |
| - | YQRGSSMVLF |
| - | RTLKRLGMDG |
| - | LGMDGYRGIS |
| - | YRGISLANWM |
| - | CLAKWESGYN |
| - | ESGYNTRATN |
| - | TRATNYNAGD |
| - | YNAGDRSTDY |
| - | RSTDYGIFQI |
| - | GIFQINSRYW |
| - | NSRYWCNDGK |
| - | CNDGKTPGAV |
| - | TPGAVNACHL |
| - | NACHLSCSAL |
| - | LQDNIADAVA |
| - | ADAVACAKRV |
| - | CAKRVVRDPQ |
| - | VRDPQGIRAW |
| - | GIRAWVAWRN |
| - | VAWRNRCQNR |
| - | RCQNRDVRQY |
| - | DVRQYVQGCG |
| - | RTPKIQVYSR |
| - | QVYSRHPAEN |
| - | HPAENGKSNF |
| - | LNCYVSGFHP |
| - | SGFHPSDIEV |
| - | SDIEVDLLKN |
| - | DLLKNGERIE |
| - | GERIEKVEHS |
| - | KVEHSDLSFS |
| - | TEKDEYACRV |
| - | YACRVNHVTL |
| - | NHVTLSQPKI |
| - | TMMKFQLLKSAEEKLFAS |
| - | MLSLKESAKMFFATKELQ |
| - | KQFTLEMAFLSKALSEMK |
| - | KLAFMLKQAELSSEKTFM |
| - | FAKFASEKKLESMTLMLQ |
| - | MLTFAEFKSMELKSQLAK |
| - | ASMFEAQLSKEKKMFTLL |
| - | ELLAKSEQAKSMLFTFMK |
| - | TKFSSFALLAQKEMLKME |
| - | MFSKMAKSLFLLAEKTQE |
| - | MATLEKLMKAFESLKSFQ |
| - | MATLEALMKAFESLKSFQ |

**3.Reg33 dataset**

| seq | regions |
| --- | --- |
| LPICPGGAARCQVTLRDLFDRAVVLSHYIHNLSSEMFSEFDKRYTHGRGFITKAINSCHTSSLATPEDKEQAQQMNQKDFLSLIVSILRSWNEPLYHLVTEVRGMQEAPEAILSKAVEIEEQTKRLLEGMELIVSQVHPETKENEIYPVWSGLPSLQMADEESRLSAYYNLLHCLRRDSHKIDNYLKLLKCRIIHNNNC | 7-34  43-57 |
| CGNLSTCMLGTYTQDFNKFHTFPQTAIGVGAP | 15-20 |
| DEPPQSPWDRVKDLATVYVDVLKDSGRDYVSQFEGSALGKQLNLKLLDNWDSVTSTFSKLREQLGPVTQEFWDNLEKETEGLRQEMSKDLEEVKAKVQPYLDDFQKKWQEEMELYRQKVEPLRAELQEGARQKLHELQEKLSPLGEEMRDRARAHVDALRTHLAPYSDELRQRLAARLEALKENGGARLAEYHAKATEHLSTLSEKAKPALEDLRQGLLPVLESFKVSFLSALEEYTKKLNTQ | 1-93 |
| KNTMEHVSSSEESIISQETYKQEKNMAINPSKENLCSTFCKEVVRNANEEEYSIGSSSEESAEVATEEVKITVDDKHYQKALNEINQFYQKFPQYLQYLYQGPIVLNPWDQVKRNAVPITPTLNREQLSTSEENSKKTVDMESTEVFTKKTKLTEEEKNRLNFLKKISQRYQKFALPQYLKTVYQHQKAMKPWIQPKTKVIPYVRYL | 81-125 |
| RSFFSFLGEAFDGARDMWRAYSDMREANYIGSDKYFHARGNYDAAKRGPGGVWAAEAISDARENIQRFFGHGAEDSLADQAANEWGRSGKDPNHFRPAGLPEKY | 1-12 |
| GPTGTGESKCPLMVKVLDAVRGSPAINVAVHVFRKAADDTWEPFASGKTSESGELHGLTTEEEFVEGIYKVEIDTKSYWKALGISPFHEHAEVVFTANDSGPRRYTIAALLSPYSYSTTAVVTNPKE | 10-20 105-115 |
| GRRRSVQWCAVSQPEATKCFQWQRNMRKVRGPPVSCIKRDSPIQCIQAIAENRADAVTLDGGFIYEAGLAPYKLRPVAAEVYGTERQPRTHYYAVAVVKKGGSFQLNELQGLKSCHTGLRRTAGWNVPIGTLRPFLNWTGPPEPIEAAVARFFSASCVPGADKGQFPNLCRLCAGTGENKCAFSSQEPYFSYSGAFKCLRDGAGDVAFIRESTVFEDLSDEAERDEYELLCPDNTRKPVDKFKDCHLARVPSHAVVARSVNGKEDAIWNLLRQAQEKFGKDKSPKFQLFGSPSGQKDLLFKDSAIGFSRVPPRIDSGLYLGSGYFTAIQNLRKSEEEVAARRARVVWCAVGEQELRKCNQWSGLSEGSVTCSSASTTEDCIALVLKGEADAMSLDGGYVYTAGKCGLVPVLAENYKSQQSSDPDPNCVDRPVEGYLAVAVVRRSDTSLTWNSVKGKKSCHTAVDRTAGWNIPMGLLFNQTGSCKFDEYFSQSCAPGSDPRSNLCALCIGDEQGENKCVPNSNERYYGYTGAFRCLAENAGDVAFVKDVTVLQNTDGNNNEAWAKDLKLADFALLCLDGKRKPVTEARSCHLAMAPNHAVVSRMDKVERLKQVLLHQQAKFGRNGSDCPDKFCLFQSETKNLLFNDNTECLARLHGKTTYEKYLGPQYVAGITNLKKCSTSPLLEACEFLRK | 538-545 |
| KKRPKPGGWNTGGSRYPGQGSPGGNRYPPQGGGGWGQPHGGGWGQPHGGGWGQPHGGGWGQPHGGGWGQGGGTHSQWNKPSKPKTNMKHMAGAAAAGAVVGGLGGYMLGSAMSRPIIHFGSDYEDRYYRENMHRYPNQVYYRPMDEYSNQNNFVHDCVNITIKQHTVTTTTKGENFTETDVKMMERVVEQMCITQYERESQAYYQRGSSMVLFSSPPVILLISFLIFLIVG | 84-125 148-171 180-196 209-231 |
| QKGGSKGRLPSEFSQFPHGQKGQHYSGQKGKQQTESKGSFSIQYTYHVDANDHDQSRKSQQYDLNALHKTTKSQRHLGGSQQLLHNKQEGRDHDKSKGHFHRVVIHHKGGKAHRGTQNPSQDQGNSPSGKGISSQYSNTEERLWVHGLSKEQTSVSGAQKGRKQGGSQSSYVLQTEELVANKQQRETKNSHQNKGHYQNVVEVREEHSSKVQTSLCPAHQDKLQHGSKDIFSTQDELLVYNKNQHQTKNLNQDQQHGRKANKISYQSSSTEERRLHYGENGVQKDVSQSSIYSQTEEKAQGKSQKQITIPSQEQEHSQKANKISYQSSSTEERRLHYGENGVQKDVSQRSIYSQTEKLVAGKSQIQAPNPKQEPWHGENAKGESGQSTNREQDLLSHEQKGRHQHGSHGGLDIVIIEQEDDSDRHLAQHLNNDRNPLFT | 1-142 |
| DAEFRHDSGYEVHHQKLVFFAEDVGSNKGAIIGLMVGGVVIA | 11-42 |
| ATASRGASQAGAPQGRVPEARPNSMVVEHPEFLKAGKEPGLQIWRVEKFDLVPVPTNLYGDFFTGDAYVILKTVQLRNGNLQYDLHYWLGNECSQDESGAAAIFTVQLDDYLNGRAVQHREVQGFESATFLGYFKSGLKYKKGGVASGFKHVVPNEVVVQRLFQVKGRRVVRATEVPVSWESFNNGDCFILDLGNNIHQWCGSNSNRYERLKATQVSKGIRDNERSGRARVHVSEEGTEPEAMLQVLGPKPALPAGTEDTAKEDAANRKLAKLYKVSNGAGTMSVSLVADENPFAQGALKSEDCFILDHGKDGKIFVWKGKQANTEERKAALKTASDFITKMDYPKQTQVSVLPEGGETPLFKQFFKNWRDPDQTDGLGLSYLSSHIANVERVPFDAATLHTSTAMAAQHGMDDDGTGQKQIWRIEGSNKVPVDPATYGQFYGGDSYIILYNYRHGGRQGQIIYNWQGAQSTQDEVAASAILTAQLDEELGGTPVQSRVVQGKEPAHLMSLFGGKPMIIYKGGTSREGGQTAPASTRLFQVRANSAGATRAVEVLPKAGALNSNDAFVLKTPSAAYLWVGTGASEAEKTGAQELLRVLRAQPVQVAEGSEPDGFWEALGGKAAYRTSPRLKDKKMDAHPPRLFACSNKIGRFVIEEVPGELMQEDLATDDVMLLDTWDQVFVWVGKDSQEEEKTEALTSAKRYIETDPANRDRRTPITVVKQGFEPPSFVGWFLGWDDDYWSVDPLDRAMAELAA | 173-230 |
| AEPRQEFEVMEDHAGTYGLGDRKDQGGYTMHQDQEGDTDAGLKESPLQTPTEDGSEEPGSETSDAKSTPTAEDVTAPLVDEGAPGKQAAAQPHTEIPEGTTAEEAGIGDTPSLEDEAAGHVTQEPESGKVVQEGFLREPGPPGLSHQLMSGMPGAPLLPEGPREATRQPSGTGPEDTEGGRHAPELLKHQLLGDLHQEGPPLKGAGGKERPGSKEEVDEDRDVDESSPQDSPPSKASPAQDGRPPQTAAREATSIPGFPAEGAIPLPVDFLSKVSTEIPASEPDGPSVGRAKGQDAPLEFTFHVEITPNVQKEQAHSEEHLGRAAFPGAPGEGPEARGPSLGEDTKEADLPEPSEKQPAAAPRGKPVSRVPQLKARMVSKSKDGTGSDDKKAKTSTRSSAKTLKNRPCLSPKHPTPGSSDPLIQPSSPAVCPEPPSSPKYVSSVTSRTGSSGAKEMKLKGADGKTKIATPRGAAPPGQKGQANATRIPAKTPPAPKTPPSSGEPPKSGDRSGYSSPGSPGTPGSRSRTPSLPTPPTREPKKVAVVRTPPKSPSSAKSRLQTAPVPMPDLKNVKSKIGSTENLKHQPGGGKVQIINKKLDLSNVQSKCGSKDNIKHVPGGGSVQIVYKPVDLSKVTSKCGSLGNIHHKPGGGQVEVKSEKLDFKDRVQSKIGSLDNITHVPGGGNKKIETHKLTFRENAKAKTDHGAEIVYKSPVVSGDTSPRHLSNVSSTGSIDMVDSPQLATLADEVSASLAKQGL | 589-600 622-627 |
| KCNTATCATQRLANFLVHSSNNFGAILSSTNVGSNTY | 8-37 |
| FGIPCCPVHLKRLLIVVVVVVLIVVVIVGALLMGL | 9-34 |
| MDVFMKGLSKAKEGVVAAAEKTKQGVAEAAGKTKEGVLYVGSKTKEGVVHGVATVAEKTKEQVTNVGGAVVTGVTAVAQKTVEGAGSIAAATGFVKKDQLGKNEEGAPQEGILEDMPVDPDNEAYEMPSEEGYQDYEPEA | 35-44 49-82 86-95 |
| KVFERCELARTLKRLGMDGYRGISLANWMCLAKWESGYNTRATNYNAGDRSTDYGIFQINSRYWCNDGKTPGAVNACHLSCSALLQDNIADAVACAKRVVRDPQGIRAWVAWRNRCQNRDVRQYVQGCGV | 5-14 25-34 56-61 |
| IQRTPKIQVYSRHPAENGKSNFLNCYVSGFHPSDIEVDLLKNGERIEKVEHSDLSFSKDWSFYLLYYTEFTPTEKDEYACRVNHVTLSQPKIVKWDRDM | 21-31 33-41 59-71 83-89 91-96 |
| RLDKQGNFNAWVAGSYGNDQWLQVDLGSSKEVTGIITQGARNFGSVQFVA | 32-50 |
| HPLGSPGSASDLETSGLQEQRNHLQGKLSELQVEQTSLEPLQESPRPTGVWKSREVATEGIRGHRKMVLYTLRAPRSPKMVQGSGCFGRKMDRISSSSGLGCKVLRRH | 66-72 |
| TQQPQQDEMPSPTFLTQVKESLSSYWESAKTAAQNLYEKTYLPAVDEKLRDLYSKSTAAMSTYTGIFTDQVLSVLKGEE | 60-70 |
| APLIPQRLMSASNSNELLLNLNNGQLLPLQLQGPLNSWIPPFSGILQQQQQAQIPGLSQFSLSALDQFAGLLPNQIPLTGEASFAQGAQAGQVDPLQLQTPPQTQPGPSHVMPYVFSFKMPQEQGQMFQYYPVYMVLPWEQPQQTVPRSPQQTRQQQYEEQIPFYAQFGYIPQLAEPAISGGQQQLAFDPQLGTAPEIAVMSTGEEIPYLQKEAINFRHDSAGVFMPSTSPKPSTTNVFTSAVDQTITPELPEEKDKTDSLREP | 112-157 |
| SSPGKPPRLVGGPMDASVEEEGVRRALDFAVGEYNKASNDMYHSRALQVVRARKQIVAGVNYFLDVELGRTTCTKTQPNLDNCPFHDQPHLKRKAFCSFQIYAVPWQGTMTLSKSTCQDA | 98-103 |
| FVNQHLCGSHLVEALYLVCGERGFFYTPKT | 11-17 |
| GIVEQCCTSICSLYQLENYCN | 13-18 |
| LIVTQTMKGLDIQKVAGTWYSLAMAASDISLLDAQSAPLRVYVEELKPTPEGDLEILLQKWENGECAQKKIIAEKTKIPAVFKIDALNENKVLVLDTDYKKYLLFCMENSAEPEQSLACQCLVRTPEVDDEALEKFDKALKALPMHIRLSFNPTQLEEQCHI | 11-20 101-110 116-126 146-152 |
| STAQSLKSVDYEVFGRVQGVCFRMYTEDEARKIGVVGWVKNTSKGTVTGQVQGPEDKVNSMKSWLSKVGSPSSRIDRTNFSNEKTISKLEYSNFSIRY | 16-31 87-98 |
| GKGDPKKPRGKMSSYAFFVQTCREEHKKKHPDASVNFSEFSKKCSERWKTMSAKEKGKFEDMAKADKARYEREMKTYIPPKGETKKKFKDPNAPKRPPSAFFLFCSEYRPKIKGEHPGLSIGDVAKKLGEMWNNTAADDKQPYEKKAAKLKEKYEKDIAAYRAKGKPDAAKKGVVKAEKSKKKKEEEDDEEDEEDEEEEEEEEDEDEEEDDDDE | 12-27 |
| MLEGKVKWFNSEKGFGFIEVEGQDDVFVHFSAIQGEGFKTLEEGQAVSFEIVEGNRGPQAANVTKEA | 1-67 |
| GPAKSPYQLVLQHSRLRGRQHGPNVCAVQKVIGTNRKYFTNCKQWYQRKICGKSTVISYECCPGYEKVPGEKGCPAALPLSNLYETLGVVGSTTTQLYTDRTEKLRPEMEGPGSFTIFAPSNEAWASLPAEVLDSLVSNVNIELLNALRYHMVGRRVLTDELKHGMTLTSMYQNSNIQIHHYPNGIVTVNCARLLKADHHATNGVVHLIDKVISTITNNIQQIIEIEDTFETLRAAVAASGLNTMLEGNGQYTLLAPTNEAFEKIPSETLNRILGDPEALRDLLNNHILKSAMCAEAIVAGLSVETLEGTTLEVGCSGDMLTINGKAIISNKDILATNGVIHYIDELLIPDSAKTLFELAAESDVSTAIDLFRQAGLGNHLSGSERLTLLAPLNSVFKDGTPPIDAHTRNLLRNHIIKDQLASKYLYHGQTLETLGGKKLRVFVYRNSLCIENSCIAAHDKRGRYGTLFTMDRVLTPPMGTVMDVLKGDNRFSMLVAAIQSAGLTETLNREGVYTVFAPTNEAFRALPPRERSRLLGDAKELANILKYHIGDEILVSGGIGALVRLKSLQGDKLEVSLKNNVVSVNKEPVAEPDIMATNGVVHVITNVLQPPANRPQERGDELADSALEIFKQASAFSRASQRSVRLAPVYQKLLERMKH | 492-509 |
| GLSDGEWQQVLNVWGKVEADIAGHGQEVLIRLFTGHPETLEKFDKFKHLKTEAEMKASEDLKKHGTVVLTALGGILKKKGHHEAELKPLAQSHATKHKIPIKYLEFISDAIIHVLHSKHPGDFGADAQGAMTKALELFRNDIAAKYKELGFQG | 1-29 101-118 |
| NEKRLVLCAASLIDSRKPLPKDGYLTIRADTFAEVFGIDVKHAYAALDDAATKLFNRDIRRYVKGKVVERMRWVFHVKYREGQGCVELGFSPTIIPHLTMLHKEFTSYQLKQIGSLSSFYAVRLYELMSQFIKLKQRECTLAQLREMFDLGDKYQDVKDMRKRVLYPALEEVNKNTDLTVAVEPRRQGRRIIGFSFTIAKNDQLALSLE | 5-13 |
| MSDSNQGNNQQNYQQYSQNGNQQQGNNRYQGYQAYNAQAQPAGGYYQNYQGYSGYQQGGYQQYNPDAGYQQQYNPQGGYQQYNPQGGYQQQFNPQGGRGNYKNFNYNNNLQGYQAGFQPQSQGMSLNDFQKQQKQAAPKPKKTLKLVSSSGIKLANATKKVGTKPAESDKKEEEKSAETKEPTKEPTKVEEPVKKEEKPVQTEEKTEEKSELPKVEDLKISESTHNTNNANVTSADALIKEQEEEVDDEVVNDMFGGKDHVSLIFMGHVDAGKSTMGGNLLYLTGSVDKRTIEKYEREAKDAGRQGWYLSWVMDTNKEERNDGKTIEVGKAYFETEKRRYTILDAPGHKMYVSEMIGGASQADVGVLVISARKGEYETGFERGGQTREHALLAKTQGVNKMVVVVNKMDDPTVNWSKERYDQCVSNVSNFLRAIGYNIKTDVVFMPVSGYSGANLKDHVDPKECPWYTGPTLLEYLDTMNHVDRHINAPFMLPIAAKMKDLGTIVEGKIESGHIKKGQSTLLMPNKTAVEIQNIYNETENEVDMAMCGEQVKLRIKGVEEEDISPGFVLTSPKNPIKSVTKFVAQIAIVELKSIIAAGFSCVMHVHTAIEEVHIVKLLHKLEKGTNRKSKKPPAFAKKGMKVIAVLETEAPVCVETYQDYPQLGRFTLRDQGTTIAIGKIVKIAE | 7-13 |
| MMNNNGNQVSNLSNALRQVNIGNRNSNTTTDQSNINFEFSTGVNNNNNNNSSSNNNNVQNNNSGRNGSQNNDNENNIKNTLEQHRQQQQAFSDMSHVEYSRITKFFQEQPLEGYTLFSHRSAPNGFKVAIVLSELGFHYNTIFLDFNLGEHRAPEFVSVNPNARVPALIDHGMDNLSIWESGAILLHLVNKYYKETGNPLLWSDDLADQSQINAWLFFQTSGHAPMIGQALHFRYFHSQKIASAVERYTDEVRRVYGVVEMALAERREALVMELDTENAAAYSAGTTPMSQSRFFDYPVWLVGDKLTIADLAFVPWNNVVDRIGINIKIEFPEVYKWTKHMMRRPAVIKALRGE | 1-89 |
| MSEPFGIVAGALNVAGLFNNCVDCFEYVQLGRPFGRDYERCQLRLDIAKARLSRWGEAVKINDDPRFHSDAPTDKSVQLAKSIVEEILLLFESAQKTSKRYELVADQQDLVVFEDKDMKPIGRALHRRLNDLVSRRQKQTSLAKKTAWALYDGKSLEKIVDQVARFVDELEKAFPIEAVCHKLAEIEIEEVEDEASLTILKDAAGGIDAAMSDAAAQKIDAIVGRNSAKDIRTEERARVQLGNVVTAAALHGGIRISDQTTNSVETVVGKGESRVLIGNEYGGKGFWDN | 218-289 |
